# Protective Effect of Ginsenoside Rg1 on Hematopoietic Stem/Progenitor Cells through Attenuating Oxidative Stress and the Wnt/β-Catenin Signaling Pathway in a Mouse Model of d-Galactose-induced Aging

**DOI:** 10.3390/ijms17060849

**Published:** 2016-06-09

**Authors:** Jing Li, Dachuan Cai, Xin Yao, Yanyan Zhang, Linbo Chen, Pengwei Jing, Lu Wang, Yaping Wang

**Affiliations:** 1 Laboratory of Stem Cells and Tissue Engineering, Chongqing Medical University, Chongqing 400016, China; jingli2523@gmail.com (J.L.); cqzyi531@gmail.com (Y.Z.); cqcb9866@outlook.com (L.C.); xhjke0316@gmail.com (P.J.); wanglu99@sina.com (L.W.); 2 Department of Pathophysiology, Chongqing Medical University, Chongqing 400016, China; moliliuxiang@aliyun.com; 3 Department of Infectious Diseases, the Second Affiliated Hospital, Chongqing Medical University, Chongqing 400016, China; cqmucdc@sohu.com; 4 Department of Histology and Embryology, Chongqing Medical University, Chongqing 400016, China

**Keywords:** ginsenoside Rg1, hematopoietic stem/progenitor cell (HSC/HPC), Wnt/β-catenin, oxidative stress, cellular senescence, d-galactose

## Abstract

Stem cell senescence is an important and current hypothesis accounting for organismal aging, especially the hematopoietic stem cell (HSC). Ginsenoside Rg1 is the main active pharmaceutical ingredient of ginseng, which is a traditional Chinese medicine. This study explored the protective effect of ginsenoside Rg1 on Sca-1^+^ hematopoietic stem/progenitor cells (HSC/HPCs) in a mouse model of d-galactose-induced aging. The mimetic aging mouse model was induced by continuous injection of d-gal for 42 days, and the C57BL/6 mice were respectively treated with ginsenoside Rg1, Vitamin E or normal saline after 7 days of d-gal injection. Compared with those in the d-gal administration alone group, ginsenoside Rg1 protected Sca-1^+^ HSC/HPCs by decreasing SA-β-Gal and enhancing the colony forming unit-mixture (CFU-Mix), and adjusting oxidative stress indices like reactive oxygen species (ROS), total anti-oxidant (T-AOC), superoxide dismutase (SOD), glutathione peroxidase (GSH-px) and malondialdehyde (MDA). In addition, ginsenoside Rg1 decreased β-catenin and c-Myc mRNA expression and enhanced the phosphorylation of GSK-3β. Moreover, ginsenoside Rg1 down-regulated advanced glycation end products (AGEs), 4-hydroxynonenal (4-HNE), phospho-histone H2A.X (r-H2A.X), 8-OHdG, p16^Ink4a^, Rb, p21^Cip1/Waf1^ and p53 in senescent Sca-1^+^ HSC/HPCs. Our findings indicated that ginsenoside Rg1 can improve the resistance of Sca-1^+^ HSC/HPCs in a mouse model of d-galactose-induced aging through the suppression of oxidative stress and excessive activation of the Wnt/β-catenin signaling pathway, and reduction of DNA damage response, p16^Ink4a^-Rb and p53-p21^Cip1/Waf1^ signaling.

## 1. Introduction

The hematopoietic stem cell (HSC) is the earliest ancestor of all blood cells, which has the ability to self-renew and has multi-directional differentiation. In the case of HSC aging, it will lead to the recession of hematopoiesis and immune function, increased incidence of tumors, difficulty in repair of tissues and organ injury, and is associated with age-related diseases [1]. Hematopoietic stem/progenitor cells (HSC/HPCs) mostly exist in bone marrow. Self renewal and multilineage differentiation ability must maintain a strict balance in order to maintain the hematopoietic stem cell pool and long-term blood cell production. Experimental studies show that hematopoietic stem cell function will decline with aging [2]. As a result, delaying hematopoietic stem/progenitor cell senescence has become a focus of geriatric medicine.

**Figure 1:**
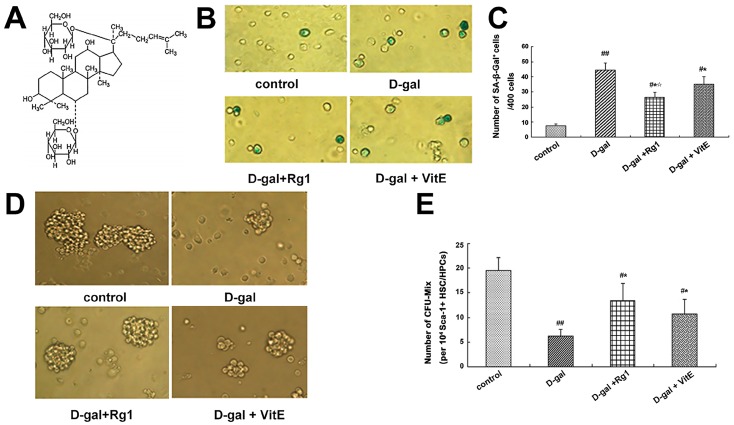
Effects of ginsenoside Rg1 on SA-β-Gal staining and CFU-Mix formation of Sca-1^+^ HSC/HPCs in a mouse model of d-gal-induced aging. (**A**) The chemical structure of ginsenoside-Rg1; (**B**) SA-β-Gal staining was performed after Sca-1^+^ HSC/HPCs were collected in different mouse model groups (×400). The aged cells were enlarged in shape and stained blue-green in the cytoplasm; (**C**) Quantification of SA-β-Gal–positive cells is shown. The number of SA-β-Gal–positive cells was decreased in the d-gal + Rg1 and d-gal + VitE groups compared to the d-gal model group. The total number of SA-β-Gal–positive cells was counted among 400 random cells; (**D**) CFU-Mix culture of Sca-1^+^ HSC/HPCs in different mouse model groups is shown (×400). In the d-gal model group, the number of CFU-Mix colonies and the number of cells that formed colonies was the lowest; (**E**) Quantification of the CFU-Mix is shown. the CFU-Mix was counted among the 1 × 10^4^ cells per hole. The CFU-Mix number in the d-gal + Rg1 group was higher than in the d-gal model group (mean ± SD, *n* = 5; ^#^ *p* < 0.05, ^##^ *p* < 0.01 *vs.* the control group, * *p* < 0.05 *vs.* the d-gal model group, ^☆^ *p* < 0.05 *vs.* the d-gal + VitE group).

Ginseng is a very famous traditional Chinese medicine. In more than 30 confirmed ginsenosides, ginsenoside Rg1 is regarded as the main active ingredient responsible for many pharmacological functions (Figure 1A) that have been widely used in clinical treatment or adjuvant treatment of many diseases. Previous experiments have confirmed the roles of ginsenoside Rg1 in improving immunity, anti-oxidation, anti-aging, enhanced memory, restoration of function and so on [3,4,5,6]. Our previous research studies have demonstrated that ginsenoside Rg1 may regulate the cell cycle and protein expression [7], inhibit telomere DNA damage and improve the activity of telomerase to delay aging. However, the mechanism through which ginsenoside Rg1 regulates the signaling pathway and its relationship with HSC/HPCs aging were not reported.

At present, the d-gal animal model has become an internationally recognized aging animal model and has been widely used in anti-aging medical research [8]. d-galactose (d-gal) is a reducing sugar that is metabolized at a normal concentration in the body. But an excess of d-gal under the action of galactose oxidase generates aldohexose and hydrogen peroxide and promotes the generation of oxygen-derived free radicals and the superoxide anion that damage the function of macromolecules and cells [9]. Some reports [10,11] demonstrated that in mice continuously exposed to d-gal, oxidative stress could be increased; this was manifested by the augmentation of reactive oxygen species (ROS) and the decline in antioxidant defense enzymes such as superoxide dismutase (SOD). Oxidative stress is one of the reasons that the body gradually ages. Additionally, some studies have suggested changes in some tissues or cells in a mouse model of d-galactose-induced aging, such as the auditory cortex [12], ventral cochlear nucleus [13], adipose-derived stem cells [14], rat hippocampus [15], *etc*. Nevertheless, there are few reports on the changes in hematopoietic stem/progenitor cells in d-galactose-induced aging mouse.

The Wnt signaling pathway has been shown to be vital for many physiological and pathophysiological processes including cell fate determination, degenerative diseases and tumorigenesis. The canonical pathway is activated through Wnt proteins connected to the receptors complex on the cell membrane consisting of Frizzled (FZD) and the low-density lipoprotein receptor-related protein 5/6 (LRP5/6), which activates the cytoplasmic protein Dishevelled (Dvl). Dvl can restrict the formation of the β-catenin destruction complex, which consists of axin, glycogen synthase kinase 3β (GSK3β) and adenomatous polyposis coli (APC), and it can also accelerate the phosphorylation of GSK-3β to become inactivate. Consequently, there will be considerable unphosphorylated β-catenin in the cytoplasm, which transfers to the nucleus and combines with the transcription T cell factor (TCF)/lymphoid enhancer factor (LEF), after which the Wnt downstream target gene transcription is activated, such as c-Myc, Cox-2 and so on. Recent experimental studies have shown that the Wnt/β-catenin signaling pathway is closely related to cellular senescence. Hongjun Liu *et al.* [16] reported that continuous Wnt exposure accelerated the aging of cells both *in vivo* and *in vitro*. Atsuhiko *et al.* [17] also indicated that complement C1q induced the activation of canonical Wnt signaling to promote an age-related phenotype. Other studies have indicated that the activation of the Wnt/β-catenin signaling pathway could give rise to cell senescence or dysfunction, such as in thymocytes [18], pulmonary epithelial cells [19], artery endothelial cells [20], muscle stem cell [21], mesenchymal stem cells [22], and intervertebral disc cells [23]. However, the relationship between the premature senescence of hematopoietic stem cells, the effects of ginsenoside Rg1 and the Wnt/β-catenin signaling pathway remain unclear.

In the current study, we investigated age-related indicators, oxidative stress indices, the related protein and gene expressions of Wnt/β-catenin signaling, and the senescence-associated protein and gene to reveal whether Rg1 can protect Sca-1^+^ HSC/HPCs in an aging mouse model induced by d-gal, its related signaling pathways and other molecular mechanisms, and the relationship between the effects of Rg1 on Sca-1^+^ HSC/HPCs aging, oxidative stress and Wnt/β-catenin signaling.

## 2. Results

### 2.1. The Effect of Ginsenoside Rg1 on the Sca-1^+^ HSC/HPCs Aging from d-Gal Administration

The mice with d-gal administration showed obvious characteristics of aging such as spirit atrophy, lags in response, drumble, withered and lackluster yellowish white fur. The proportion of Sca-1^+^ HSC/HPCs was 9.17% ± 1.06% in the mouse bone marrow-derived mononuclear cells before the purification procedure. Hence, in order to extract Sca-1^+^ HSC/HPCs for further research, the mouse bone marrow cells from the different treatment model groups were isolated and purified by MACS. The purity of the Sca-1^+^ HSC/HPCs was determined to be 90.87% ± 2.3%, and the survival rate of the separated cells was 98.2% ± 1.4% according to the Trypan blue dye exclusion assay.

Sa-β-gal staining is one of the methods that is widely used to determine cell aging [24]. Therefore, SA-β-gal staining was performed to observe the effects of Rg1 on Sca-1^+^ HSC/HPCs in a mouse model of d-gal-induced aging. A blue-green color was observed in the cytoplasm of positive cells and no color was observed in negative cells (Figure 1B). Compared to the control group, the percentage of Sa-β-gal positive cells in the d-gal model group increased significantly; in the d-gal + Rg1 and d-gal + VitE groups, the percentage of Sa-β-gal positive cells decreased significantly compared to those in the d-gal model group (* *p* < 0.05), but the percentage of positive cells with Sa-β-gal dye in the d-gal + Rg1 group was lower than in the d-gal + VitE group (^☆^ *p* < 0.05).

The Mix colony-forming capacity can react with the multi-directional differentiation properties of HSCs. As the HSCs age, the capacity to form CFU-Mix is gradually reduced. As shown in Figure 1D, compared with that of the control group, there were much fewer CFU-Mix colonies and much fewer cells in each colony in the d-gal model group (^##^ *p* < 0.01). However, in the d-gal + Rg1 group and d-gal + VitE group, the number of CFU-Mix colonies was increased compared to the d-gal model group.

### 2.2. The Anti-Oxidative Stress Effects of Ginsenoside Rg1 on Sca-1^+^ HSC/HPCs Aging from d-Gal Administration

According to the free radical or oxidative stress theory of aging, oxidative stress that damages various macromolecules occurs because of the imbalances between ROS and antioxidants. ROS are chemically reactive molecules that include oxygen ions and peroxides. Therefore, the ROS and T-AOC level in cells, SOD and GSH-px activity, and MDA contents in the mice serum were evaluated to confirm whether the anti-aging effects of ginsenoside Rg1 were mediated by alleviating the oxidative stress caused by d-gal administration.

**Figure 2:**
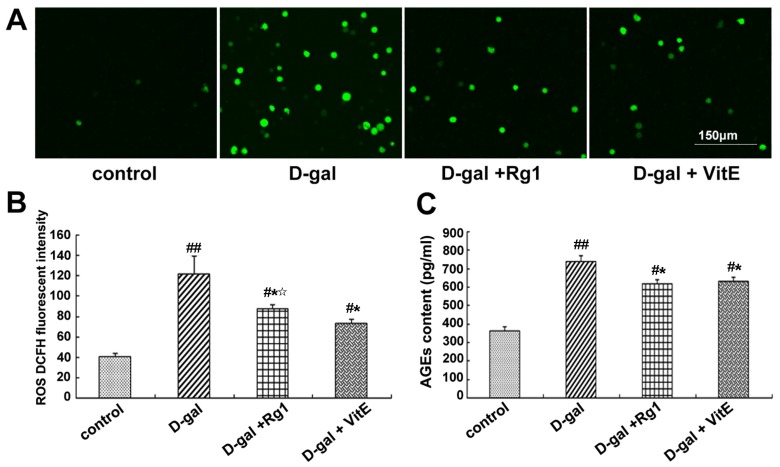
Effects of ginsenoside Rg1 on ROS level and AGEs content in the mouse model of d-gal-induced aging. (**A**) The DCFH (dichlorodi hydrofluorescein diacetate) fluorescence level of ROS is shown. In the d-gal group, more positive green fluorescence was observed through laser scanning confocal microscopy. There were fewer ROS-stained Sca-1^+^ HSC/HPCs in the d-gal + Rg1 group than in the d-gal model group. Green indicates ROS staining. Scale bar = 150 μm; (**B**) Quantification assays of the ROS level are shown. Intracellular ROS generation was detected through flow cytometry. In the d-gal model group, the DCFH fluorescence intensity was evidently higher than that in the other groups; (**C**) Effects of ginsenoside Rg1 on AGEs in aging mice serum. AGE content in the d-gal group was significantly higher compared to the other groups (mean ± SD, *n* = 5; ^#^ *p* < 0.05, ^##^ *p* < 0.01 *vs.* the control group, * *p* < 0.05 *vs.* the d-gal model group, ^☆^ *p* < 0.05 *vs.* the d-gal + VitE group).

Through the DCFH fluorescence analysis of ROS, the majority of cells in the d-gal model group displayed positive green fluorescence, whereas green fluorescence was rare in the control group (Figure 2A). In the d-gal + Rg1 and d-gal + VitE groups, the ROS level was lower than that in the d-gal model group (* *p* < 0.05).

As shown in Table 1, compared to the control group, the T-AOC level, SOD and GSH-px activities were significantly lower in the d-gal model group, while the MDA contents were higher. Meanwhile, ginsenoside Rg1 partially rescued the reduction of the T-AOC level, SOD and GSH-px activities, leading to a decrease in the MDA content in the d-gal + Rg1 group (* *p* < 0.05). There was no significant difference between the d-gal + Rg1 and d-gal + VitE groups except for SOD.

Advanced glycationend products (AGEs) are the binding products of excess sugar and protein, which can accelerate the body’s aging and lead to considerable chronic degeneration disease. AGEs can be used as a marker of oxidative stress and inflammation [25]. AGEs in mice treated with d-gal showed a remarkable increase compared to the control group (Figure 2C). The increased level of AGEs in d-gal-treated mice significantly reversed after ginsenoside Rg1 treatment (* *p* < 0.05). The AGE content in mice serum in the d-gal + Rg1 group was similar to that in the d-gal + VitE group.

### 2.3. Ginsenoside Rg1 Diminished the β-Catenin Expression of Sca-1^+^ HSC/HPCs in d-Gal-induced Aging Mice

**Figure 3:**
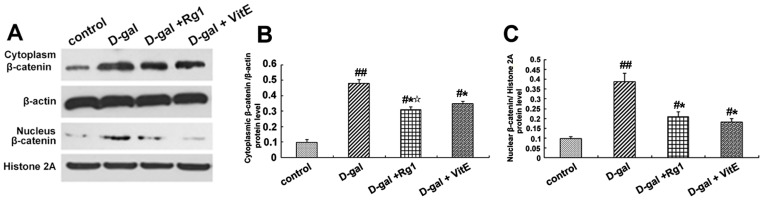

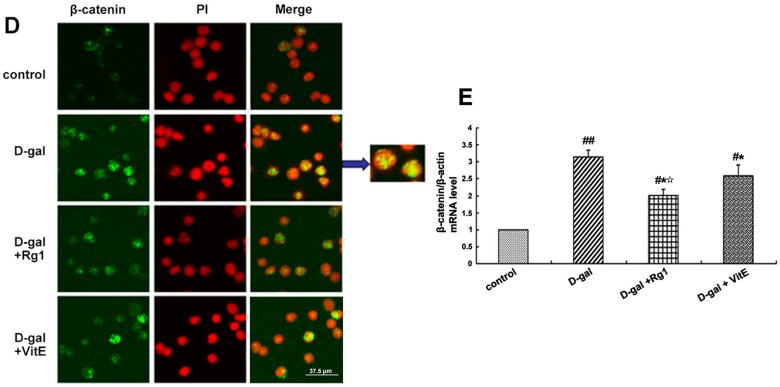
Effects of ginsenoside Rg1 on β-catenin expression of Sca-1^+^ HSC/HPCs from d-gal-induced aging mice. (**A**) The cytoplasmic and nuclear β-catenin is shown by Western blot. β-actin was used as the internal control for the cytoplasmic proteins, whereas histone 2A was the internal control for the nuclear proteins; (**B**,**C**) Quantification of cytoplasmic and nuclear β-catenin protein levels is shown. In the d-gal model group, the expressions of cytoplasmic and nuclear β-catenin were increased compared to the other three groups; (**D**) Immunofluorescence staining of β-catenin is shown. There was an obvious increase in fluorescent protein in the cell nucleus of the d-gal model group. Green indicates β-catenin mAb. Red indicates nuclei stained by PI. Scale bar = 37.5 μm; (**E**) The mRNA expression of β-catenin was assessed by real-time quantitative RT-PCR, in which β-actin was used as an internal control (mean ± SD, *n* = 3; ^#^ *p* < 0.05, ^##^ *p* < 0.01 *vs.* the control group, * *p* < 0.05 *vs.* the d-gal model group, ^☆^ *p* < 0.05 *vs.* the d-gal + VitE group).

The β-catenin expression in the Sca-1^+^HSC/HPCs was examined by Western blot analysis and immunofluorescence in order to identify the activity of Wnt/β-catenin signaling. The β-catenin expression in the cytoplasm and nucleus was detected by Western blotting. According to Figure 3A–C, compared to the control model group, the expressions of cytoplasmic and nuclear β-catenin increased in the d-gal model group. After ginsenoside Rg1 or VitE treatment, the expression of β-catenin was reduced compared with that in the aging model group (* *p* < 0.05). The expression of cytoplasmic β-catenin in the d-gal + Rg1 group was lower than that in the d-gal + VitE group.

The location expression of β-catenin was shown by immunofluorescence to further observe the nuclear translocation. In the d-gal group, there was significantly enhanced fluorescence intensity of the β-catenin protein in the Sca-1^+^ HSC/HPCs, and there was also an increase in the transfer to the nucleus. However, the treatment with Rg1 decreased the nuclear translocation of β-catenin (Figure 3D).

Furthermore, the gene expression of β-catenin was detected by RT-PCR. As shown in Figure 3E, β-catenin mRNA expression was upregulated in the d-gal model group compared to the other three groups (* *p* < 0.05, ^##^ *p* < 0.01). After treatment with ginsenoside Rg1, the β-catenin mRNA expression was clearly lower than that in the d-gal + VitE group (^☆^ *p* < 0.05).

### 2.4. The Effects of Ginsenoside Rg1 on GSK-3β and pGSK-3β of Sca-1^+^ HSC/HPCs from d-Gal-induced Aging Mice

**Figure 4:**
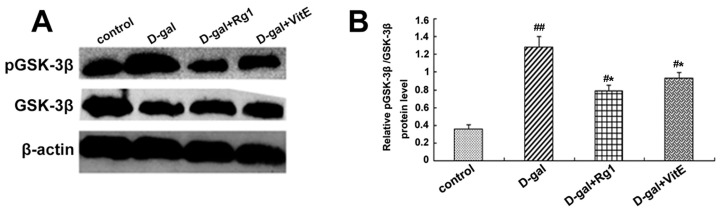

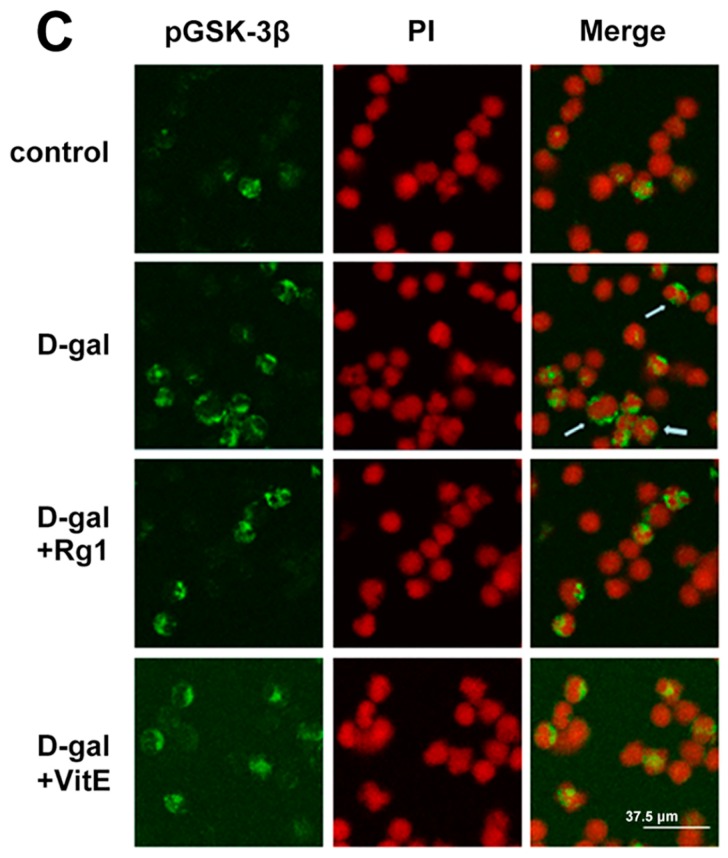
Effects of ginsenoside Rg1 on GSK-3β and pGSK-3β expressions of Sca-1^+^ HSC/HPCs from d-gal-induced aging mice. (**A**) GSK-3β and pGSK-3β expressions are shown by Western blot. β-actin was the internal control protein; (**B**) The ratio of pGSK-3β/total GSK-3β is shown. The pGSK-3β/total GSK-3β levels increased in the d-gal model group compared to the control group and d-gal + Rg1 group; (**C**) Immunofluorescence staining of pGSK-3β is shown. The pGSK-3β expression was strong in the d-gal model group compared to the control group, but was decreased after treatment with Rg1 and VitE. Green indicates pGSK-3β mAb in the cytoplasm, which was pointed out by the arrow in the diagram. Red indicates nuclei stained by PI. Scale bar = 37.5 μm. (mean ± SD, *n* = 3; ^#^ *p* < 0.05, ^##^ *p* < 0.01 *vs.* the control group, * *p* < 0.05 *vs.* the d-gal model group).

GSK-3β is a member of the β-catenin destruction compound and a negative regulator of the Wnt pathway. The phosphorylation at Ser9 [26] of GSK-3β results in loss of activity. As shown in Figure 4A,B, in the d-gal group, the GSK-3β expression had a slight decline. On the contrary, the phosphorylation of GSK-3β was markedly enhanced so that the ratio of pGSK-3β/total GSK-3β was higher compared to the control group (^##^ *p* < 0.01). However, a partial decrease in the ratio pGSK-3β/total GSK-3β was observed in the the d-gal + Rg1 and the d-gal + VitE groups.

Through immunofluorescence observation (Figure 4C), the pGSK-3β expression in the cytoplasm was found to have a significant increase in the d-gal group compared to the control group. At the same time, after treatment with ginsenoside Rg1 or VitE, the green fluorescence intensity was weaker than d-gal administration alone.

### 2.5. Ginsenoside Rg1 Decreased c-Myc mRNA, TCF-4, and 4-HNE Expressions in Sca-1^+^ HSC/HPCs from d-Gal-induced Aging Mice

**Figure 5:**
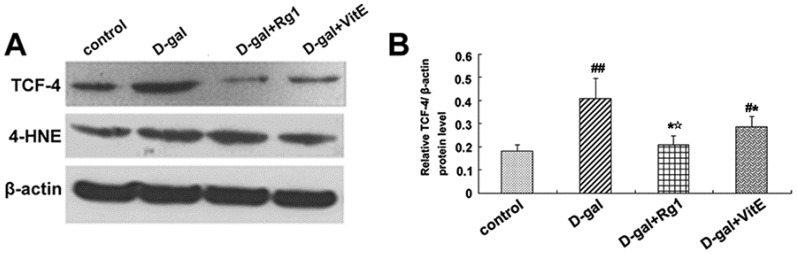

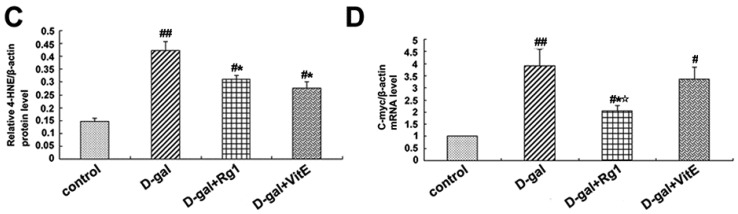
Effects of ginsenoside Rg1 on c-Myc mRNA, 4-HNE, and TCF-4 expressions of Sca-1^+^ HSC/HPCs from d-gal-induced aging mice. (**A**) TCF-4 and 4-HNE expressions are shown by Western blot analysis. β-actin was the internal control protein; (**B**,**C**) Quantification of TCF-4 and 4-HNE protein levels is shown. TCF-4 and 4-HNE protein levels were both upregulated in the d-gal model group compared to the other experimental groups (^##^ *p* < 0.01); (**D**) The c-Myc mRNA expression was detected by qRT-PCR. All values were normalized against β-actin and expressed as a percentage of the control. Data are expressed as the means ± SD. The experiments were performed three times. (^#^ *p* < 0.05, ^##^ *p* < 0.01 *vs.* the control group, * *p* < 0.05 *vs.* the d-gal model group, ^☆^ *p* < 0.05 *vs.* the d-gal + VitE group).

Through the activation of the canonical Wnt signaling pathway, cytosolic β-catenin is stabilized and translocates to the nucleus where it binds to T cell factor/lymphoid enhancer factor (TCF/LEF) and induces TCF/LEF-dependent transcription. Then, the Wnt downstream target gene transcription is also activated, such as c-Myc. In addition, 4-HNE is a kind of oxidative stress product formed during lipid peroxidation. It is present at higher levels during oxidative stress. According to Figure 5, the expressions of c-Myc mRNA, TCF-4, and 4-HNE in the d-gal group were obviously higher than in the control group (^##^ *p* < 0.01), while ginsenoside Rg1 treatment remarkably reduced the c-Myc mRNA, TCF-4, and 4-HNE expressions compared to d-gal administration alone.

### 2.6. Ginsenoside Rg1 Slowed down DNA Damage Responses in Sca-1^+^ HSC/HPCs from d-Gal-induced Aging Mice

**Figure 6:**
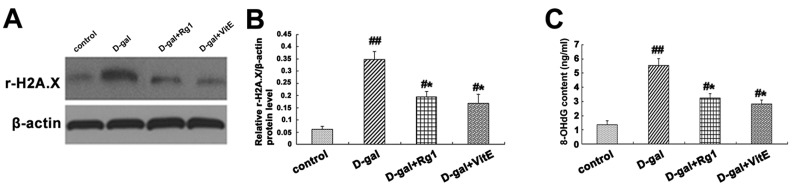

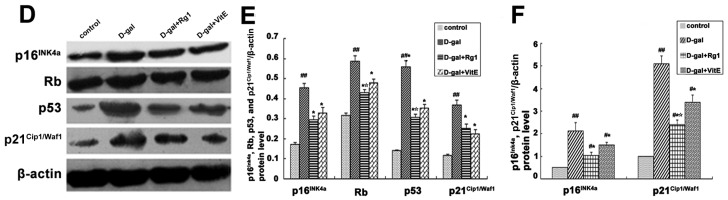
Effects of ginsenoside Rg1 on r-H2A.X, 8-OHdG, p16^Ink4a^, Rb, p53, p21^Cip1^^/Waf1^, p16^Ink4a^ mRNA, and p21^Cip1^^/Waf1^ mRNA expressions of Sca-1^+^ HSC/HPCs from d-gal-induced aging mice. (**A**) The r-H2A.X expression is shown by Western blot analysis. β-actin was the internal control; (**B**) Quantification of r-H2A.X expression is shown. The r-H2A.X expression was significantly up-regulated in the d-gal group compared with the other three groups (^##^ *p* < 0.01, * *p* < 0.05); (**C**) The 8-OHdG levels in the mice serum in each group were measured using an ELISA kit. Data are expressed as the means ± SD. The experiments were performed five times; (**D**) The p16^Ink4a^, Rb, p53, and p21^Cip1^^/Waf1^ expressions are shown by Western blot analysis. β-actin was the internal control protein; (**E**) Quantification of p16^Ink4a^, Rb, p53, and p21^Cip1^^/Waf1^ protein levels is shown. Treatment with Rg1 or VitE was found to alleviate the expressions of p16^Ink4a^, Rb, p53, and p21^Cip1^^/Waf1^ protein induced by d-gal treatment; (**F**) The p16^Ink4a^ and p21^Cip1^^/Waf1^ mRNA expressions were assessed by RT-PCR. β-actin was the internal control. The results displayed the obvious enhancement of p16^Ink4a^ and p21^Cip1^^/Waf1^ mRNA expressions in the d-gal group compared to the other groups. mean ± SD, *n* = 3. (^#^ *p* < 0.05, ^##^ *p* < 0.01 *vs.* the control group, * *p* < 0.05 *vs.* the d-gal model group, ^☆^ *p* < 0.05 *vs*. the d-gal + VitE group).

DNA damage response (DDR) is one of the main causes of senescence in multicellular organism [27]. The expression of r-H2A.X is an important marker for the formation of DNA damage foci. The up-regulation of r-H2A.X expression occurred in the d-gal model group compared to the control groups. Meanwhile, after adding the Rg1 or VitE treatments, the r-H2A.X expression levels were significantly lower than those in the d-gal group (Figure 6A,B).

8-Hydroxy-2′-deoxyguanosine (8-OHdG) is another biomarker for DNA damage, formed when DNA is oxidatively modified by ROS, which is one of the most sensitive biological symbols for oxidative stress that can be detected in various biological sample [28]. As Figure 6C shows, the 8-OHdG level in mice serum detected by ELISA was markedly higher in the d-gal model group than in the control group. However, the 8-OHdG level declined substantially in the d-gal + Rg1 or d-gal + VitE group (* *p* < 0.05).

### 2.7. Ginsenoside Rg1 Down-Regulated the Expressions of p16^Ink4a^, Rb, p53, and p21^Cip1^^/Waf1^ in Sca-1^+^ HSC/HPCs from d-Gal-induced Aging Mice

The p16^Ink4a^-Rb and p19^Arf^-Mdm2-p53-p21^Cip1/Waf1^ pathways play an important role in the induction of cell aging process. As shown in Figure 6D,E, in the d-gal model group, p16^Ink4a^, Rb, p53, and p21^Cip1/Waf1^ protein expressions were enhanced compared to the control group, whereas the d-gal + Rg1 group showed a clear decrease in these expressions (* *p* < 0.05). The p16^Ink4a^ and p21^Cip1/Waf1^ mRNA expressions in the d-gal model group were significantly higher, while treatment with Rg1 or VitE resulted in down-regulated expressions of the genes (Figure 6F).

## 3. Discussion

Hematopoietic stem cells (HSCs) are unable to escape the harmful effect of the aging process. Molecular mechanisms that are associated with aging mainly include telomere dysfunction, mitochondrial metabolism disorder, and cellular senescence, inflammation, as well as altered signal pathways [29,30,31]. How to postpone hematopoietic stem cell aging has become a hot topic for human beings.

d-Gal was injected into the mice continuously within a certain time, which will lead to an increase in the concentration of intracellular galactose *in vivo*, significantly increasing the level of ROS, causing oxidative stress and inducing aging in animals [13,32]. In our present research, the corresponding changes in cell aging-related indexes took place in Sca-1^+^ HSC/HPCs from d-gal-induced aging mice. SA-β-Gal, which reflects the lysosomal function, accumulates significantly in the cytoplasm with cell aging and lysosome expansion [33]. CFU-Mix is also one of the widely used biological indices to judge HSC/HPCs aging [34]. In the mice model of d-gal administration alone, the number of SA-β-Gal positive Sca-1^+^ HSC/HPCs were significantly increased (Figure 1B) and the number of CFU-Mix and colony forming cells declined dramatically (Figure 1D). However, after treatment with Rg1, the increased positive rate of SA-β-Gal was reversed, and the formation of CFU-Mix increased to some degree. These results suggest that ginsenoside Rg1 had protective effects with respect to Sca-1^+^ HSC/HPCs aging induced by d-gal administration *in vivo*.

The free radical damage theory indicates that the generation of free radicals or ROS can result in cell and tissue damage as well as changes in the function of the gene device, leading to aging and premature cell death. Total antioxidant capacity (T-AOC) can reflect the body’s overall resistance to free radicals from the enzymatic and non-enzymatic defense systems [35], which can measure the body index of anti-aging. SOD and GSH-px are the most important antioxidant enzymes that also act as the original protection system against ROS that are generated during oxidative stress *in vivo* [36]. Malondialdehyde (MDA) is formed in the lipid peroxidation caused by ROS and is also used as a biomarker to measure the level of oxidative stress in an organism. In the present study, there were clear increases in the ROS concentration and the decline of T-AOC capacity in Sca-1^+^ HSC/HPCs from d-gal-treated mice, a reduction in SOD and GSH-px activity, and an increase in the MDA contents in d-gal-treated mice serum (Table 1). Nevertheless, ginsenoside Rg1 offset the changes of these biomarkers induced by d-gal.

In addition, the expression of 4-HNE, 8-OHdG and AGEs also significantly increased in the d-gal model group. Aging has a close relationship with increases in the levels of endogenous ROS and decreases in antioxidant defenses, resulting in extensive oxidative stress damage in cell structures, including membrane lipid peroxidation, enzyme inactivation, protein oxidation, and DNA oxidative damage [37]. 4-HNE is an indicator of lipid peroxidation and protein lesions, and is considered a second toxic messenger of ROS [38]. 8-OHdG is a major oxidative base damage in DNA or nucleotides and is induced by ROS [39]. However, ginsenoside Rg1 treatment reduced the expression of 4-HNE and 8-OHdG, which indicates that the protective effect of ginsenoside Rg1 on lipid peroxidation and DNA oxidative damage induced by d-gal. Advanced glycation end products (AGEs) are a group of heterogenous molecules [40] and a trigger of excessive ROS and abnormally high oxidative stress that accumulate in the body with age and are thus considered as biomarkers of senescence [41]. Our results demonstrated that ginsenoside Rg1 treatment evidently alleviated the elevated level of AGEs. Therefore, through testing the above related parameters of oxidative stress, ginsenoside Rg1 was proved to show active antioxidant effects in Sca-1^+^ HSC/HPCs and the body of mice to prevent d-gal-induced oxidative damage of tissues and cells, as well as reverse the aging phenotype. Furthermore, its role was not inferior to that of the well-known VitE.

Previous studies have shown that the Wnt/β-catenin signaling pathway is crucial to stem cell fate determinations such as stem cell differentiation, proliferation, apoptosis and aging [42,43]. We examined the correlation indices of the Wnt/β-catenin signaling pathway. β-catenin, the core protein of the canonical Wnt signaling pathway, was apparently accumulated both in the cytoplasm and nuclei in Sca-1^+^ HSC/HPCs from d-gal-administrated mice alone. However, after treatment with ginsenoside Rg1, the protein and gene expressions of β-catenin were significantly down-regulated in Sca-1^+^ HSC/HPCs (Figure 3). It turned out that the Wnt/β-catenin signaling pathway was activated in Sca-1^+^ HSC/HPCs from d-gal-treated mice and that the protective effects of Rg1 are related to the Wnt signaling pathway. GSK-3β negatively regulates β-catenin by sequestration and promotion of its proteosome degradation. The phosphorylation of GSK-3β at Ser-9 will mediate its inactivation [26]. Upon inactivation, its inhibitory impact on β-catenin is relieved, and β-catenin is allowed to accumulate and translocate to the nucleus. There was an obvious increase in the pGSK-3β fluorescence intensity in the cytoplasm and the pGSK-3β/total GSK-3β ratio in Sca-1^+^ HSC/HPCs from d-gal-treated mice (Figure 4). On the contrary, the down-regulation of pGSK-3β was detected in the d-gal + Rg1 group, showing that Rg1 alleviated the inactivation of GSK-3β and the subsequent accumulation of β-catenin in Sca-1^+^ HSC/HPCs from d-gal-induced aging mice.

In the canonical Wnt signaling, β-catenin subsequently translocated into the nucleus to activate the TCF/LEF transcription factor, which activated the Wnt target genes. After treatment with Rg1, the TCF-4 expression was decreased compared to that in the d-gal model group, indicating that Rg1 counteracted the increased transcriptional activity in Sca-1^+^ HSC/HPCs from d-gal-induced aging mice. One of the target genes of the Wnt signaling pathway is Proto-oncogene c-Myc [44]. Recent research has shown that c-Myc over-expression can also induce cell senescence because c-Myc is associated with DNA damage response [45] and increased ROS [46]. In the d-gal model group, the up-regulation of c-Myc mRNA expression in Sca-1^+^ HSC/HPCs further confirmed the activation of the Wnt signaling pathway and the promotion of cell senescence. However, Rg1 treatment reduced c-Myc mRNA expression to a certain extent to slow down cell aging (Figure 5). As a consequence, these above results demonstrated that ginsenoside Rg1 attenuated the activation of the Wnt signaling pathway induced by d-gal, which agreed with some previous studies of ginsenoside, confirmed the action mechanism of ginsenoside might be relevant to Wnt signaling [47,48], e.g., that ginsenoside Rg3 could lead to the down-regulation of Wnt/β-catenin signaling in colorectal cancer [49].

Meanwhile, to further probe the relationship between oxidative stress and the Wnt signaling pathway, Vitamin E (VitE) was adopted as positive control. VitE is a kind of lipid-soluble vitamin, with oxidation resistance and many experiments established a role in regulating signaling pathways [50]. After VitE treatment, ROS scavenging, decreased β-catenin, pGSK-3β, TCF-4 protein levels and c-Myc mRNA amplification were observed compared to d-gal administration alone. These studies have demonstrated that Sca-1^+^ HSC/HPCs aging from d-gal administration was implicated in the oxidative stress-mediated Wnt/β-catenin signaling pathway. This result was supported by previous studies that found that mouse extraembryonic endoderm patterns might be affected by the crosstalk oxidative stress and the Wnt/β-catenin signaling pathway [51]. Furthermore, ROS signaling in the form of H_2_O_2_ separated nucleoredoxin (Nrx) from Dvl, thereby activating canonical Wnt/β-catenin signaling [52]. Therefore, ginsenoside Rg1 also decreased ROS generation and inhibited the activation of the Wnt/β-catenin signaling. Ginsenoside Rg1 improved the resistance of Sca-1^+^ HSC/HPCs to d-gal-induced senescence *in vivo* through the oxidative stress-mediated Wnt/β-catenin signaling pathway, at least partly. It is possible that there are other mechanisms, and this requires further study.

It is well-known that the DNA damage response (DDR), p16^Ink4a^-Rb and p19^Arf^ -p53-p21^Cip1/Waf1^ pathways are important biological mechanisms in the stem cell aging process [53]. Recent experiments confirmed that in the process of aging, there was an accumulation of DNA damage, which restricted the self-renewal capacity of HSCs [54,55]. The r-H2A.X expression is an indicator of the DNA damage extent and 8-OHdG is an established indicator of DNA oxidative stress injury. The present study showed the expressions of r-H2A.X and 8-OHdG were increased in d-gal-induced mice, while they were down-regulated in different degrees after the administration with ginsenoside Rg1 or VitE (Figure 6). Furthermore, the reduced expressions of p21^Cip1/Waf1^, p53, Rb and p16^Ink4a^ induced by Rg1 demonstrated that ginsenoside Rg1 could attenuate the expressions of senescence-associated protein and genes in Sca-1^+^ HSC/HPCs from d-gal-induced aging mice. Previous research has demonstrated the accumulation of ROS could result in DNA damage [56] and the activity of p53 [57], p16^Ink4a^ [58]. In conclusion, DDR and the p16^Ink4a^-Rb and p53-p21^Cip1/Waf1^ pathways may be involved in Sca-1^+^ HSC/HPC aging from the d-gal-induced mouse model. Moreover, ginsenoside Rg1 could regulate these age-related pathways to delay Sca-1^+^ HSC/HPC aging, which may abate the oxidative stress reaction *in vivo*.

## 4. Materials and Methods

### 4.1. Animal Treatment

Male C57BL/6 mice, weight of 19–21 g, 6–8 weeks old were purchased from the Laboratory Animal Center of Chongqing Medical University, breeding conditions: 20–25 °C, natural lighting, free water and feeding. All of the experiments were performed according to the institutional regulations and approved by the Chongqing Medical University Animal Care and Use Committee ((Permit number: SCXK (Chongqing) 2013-0007), China).

Mice were divided into 4 groups at random: (1) control group; (2) d-gal model group; (3) the d-gal + Rg1 group; (4) the d-gal +VitE group. In the d-gal model group, d-gal (120 mg/kg·day) was injected subcutaneously daily into mice for 42 days. In the d-gal + Rg1 group and d-gal +VitE group, since the 8th day of d-gal injection, ginsenoside Rg1 (20 mg/kg·day) or VitE (100 mg/kg·day) was injected intraperitoneally daily for 35 days concomitantly. All the control mice were given the same amount of saline subcutaneously and in the abdominal cavity.

### 4.2. Reagents

Ginsenoside Rg1 (Purity = 98.3%, RSZD-121106) was obtained from Xi’an Haoxuan Biological Technology Co., Ltd. (Xi’an, China). d-galactose was purchased from Shanghai Puzhen biological science and Technology Co., Ltd. (Shanghai, China). Fetal bovine serum (FBS) and Iscove’s Modified Dulbecco’s Medium (IMDM) were purchased from Gibco (Waltham, MA, USA). The Anti-Sca-1^+^ Micro Bead Kit was purchased from Miltenyi Biotech Co. (Bergisch Gladbach, Germany). The SA-β-gal Staining and Reactive Oxygen Species Assay Kits were purchased from the Beyotime Institute of Biotechnology (Shanghai, China). The SOD, GSH-px, T-AOC and MDA kits were obtained from Nanjing Jiancheng Bioengineering Institute (Nanjing, China). The methylcellulose semi solid culture medium for CFU-mix was purchased from Stem Cell Technologies (Vancouver, BC, Canada). The antibodies against β-catenin, GSK-3β, Phospho-GSK-3β, TCF-4, and r-H2A.X were obtained from Cell Signaling Technology (Danvers, MA, USA). The antibodies against p16^INK4a^, Rb, p53, p21^Cip1/Waf1^, β-actin and Histone 2A were purchased from Santa Cruz (San Cruz, CA, USA), and Anti-4-HNE antibody was purchased from Abcam (Cambridge, MA, USA). The Mouse ELISA Kit for 8-OHdG or AGEs was obtained from Shanghai Yuanye Bio- Technology Co., Ltd. (Shanghai, China).

### 4.3. Isolation and Purification of Sca-1^+^ HSC/HPCs

The femur and tibia of C57BL/6 mice were taken out under aseptic conditions and the bone marrow cells were rushed out with IMDM. Red blood cells were depleted with Red Blood Cell Lysis Buffer, and then bone marrow mononuclear cells were isolated by density gradient centrifugation using mononuclear cell separation fluid (Sigma-Aldrich, St. Louis, MO, USA). Mouse bone marrow-derived Sca-1 positive HSC/HPCs were isolated by the magnetic activated cell sorting (MACS) technique as an established protocol [59,60]. Hence, the Sca-1 positive cells obtained were used for subsequent experimental measurement.

### 4.4. Senescence-Associated β-Galactosidase Cytochemical Staining

The Senescence-β-Galactosidase (SA-β-Gal) Staining Kit (Beyotime Institute of Biotechnology, Shanghai, China) was used to detect cell aging. According to the manufacturer’s instructions, 1 × 10^5^ cells were fixed for 10 min with fixative solution at room temperature, with PBS washing three times. Then, cells were incubated in SA-β-Gal–staining solution for 12 h at 37 °C at pH 6.0 without CO_2_ in darkness. The SA-β-Gal–positive cells were dyed blue-green. For quantification purposes, at least 400 cell fields were scored for staining in five random microscopy fields, and the percentage of positive cells in each group was calculated.

### 4.5. Mixed Colony-Forming Unit of Sca-1^+^ HSC/HPCs Culture

Formation of the CFU-Mix represented the pluripotent nature of Sca-1^+^ HSC/HPCs. A total of 4 × 10^4^ Sca-1^+^ HSC/HPCs from each treatment group were collected, mixed with 2 mL methyl cellulose semi-solid culture medium (MethoCult #3434, Stem Cell Technologies), then incubated in 24-well plates for 7–12 days with 5% CO_2_ at 37 °C. The number of mixed colonies was counted in triplicate holes per group.

### 4.6. Measurement of Reactive Oxygen Species (ROS) Level

The ROS level was detected by the Reactive Oxygen Species Assay Kit (Beyotime Institute of Biotechnology, Shanghai, China), whose operating principle is the use of fluorescent probes DCFH-DA to detect ROS. A total of 1 × 10^6^ Sca-1^+^ HSC/HPCs were collected in each treatment group and incubated in 2’,7’-dichlorofluorescein diacetate (DCFH-DA) with 5% CO_2_ at 37 °C for 20 min. ROS in mitochondria were determined using flow cytometry and laser scanning confocal microscopy (LSM510; Carl Zeiss, Jena, Germany).

### 4.7. Detection of Oxidative Stress-Associated Biological Indicators

The Sca-1^+^ HSC/HPCs in each treatment group were collected and lysed. The supernatant was collected after centrifugation. Following the manufacturer’s protocols, T-AOC content was detected by the assay kits (Jiancheng, Nanjing, China). The mice in each group were anesthetized and the serum was separated from heart blood by centrifugation. SOD activity, GSH-px activity, and MDA content were measured using the corresponding assay kits.

### 4.8. Determination of AGEs and 8-OHdG Concentration with ELISA

The mice serum of each model groups was separated as described above, and the levels of AGEs and 8-OHdG in each group were detected by an ELISA kit following the manufacturer’s instructions (Shanghai Yuanye Bio-Technology Co., Ltd., Shanghai, China).

### 4.9. Western Blot Analysis

The Sca-1^+^ HSC/HPCs in each treatment group were collected, and the total cell protein extracts were measured by a BCA assay, To detect β-catenin, cell proteins in the cytoplasm and nucleus were respectively extracted using the Nuclear and Cytoplasmic Protein Extraction Kit (Beyotime Institute of Biotechnology, Shanghai, China). After separated by SDS-PAGE, proteins were transferred to PVDF membranes (Millipore, Marlborough, MA, USA), which were incubated with the primary antibodies against β-catenin, GSK-3β, Phospho-GSK-3β, TCF-4, r-H2A.X (Cell Signaling Technology), p16^INK4a^, Rb, p53, p21^Cip1/Waf1^, β-actin, Histone 2A (Santa Crus), and 4-HNE (Abcam). β-actin and Histone 2A were used as the internal controls for the cytoplasmic and nuclear proteins, respectively. Signals were observed after incubation with HRP-labeled secondary antibodies (Jackson, West Grove, PA, USA) by using electro-chemi-luminescence (ECL). The semi-quantification analysis was performed using Quantity One software (BioRad, Hercules, CA, USA).

### 4.10. Immunofluorescence Staining

After Sca-1^+^ HSC/HPCs were gathered in each group, the 20–30 μL cell suspension was dropped onto the glass slide, fixed with 4% paraformaldehyde, washed with TBS, blocked with 10% goat serum, and incubated with antibodies against β-catenin (1:100) and Phospho-GSK-3β (1:100) overnight at 4 °C. Then, the cells were washed and incubated for 1 h with Cy3-labeled goat anti-rabbit IgG (1:300). The nuclei were counterstained with PI. The cells were imaged using a fluorescence microscope (LSM510; Carl Zeiss, Jena, Germany).

### 4.11. Real-Time Quantitative RT-PCR

Total RNA was extracted from Sca-1^+^ HSC/HPCs with Trizol reagent (TaKaRA, Kyoto, Japan). RNA was reverse transcribed into cDNA (TaKaRA). Real-time PCR was performed using a BIO-RAD sequence detection system (FX96) (Bio-Rad, Pleasanton, CA, USA). All experiments were carried out in triplicate and were normalized to the control gene β-actin. The PCR primers included: β-catenin Forward (5’CGTGCGCATGGAGGAGATAGTAG3’) and β-catenin Reverse (5’CCCCTGCAGCTACTCTTTGGATA3’); c-Myc Forward (5’CCCACCACCAGCAGCGACTC3’) and c-Myc Reverse (5’GCCCGACTCCGACCTCTTGG3’); p21^Cip1/Waf1^ Forward (5’TGCTCTTTTCCCCCACCCCATAC3’) and p21^Cip1/Waf1^ Reverse (5’CCCCCACCACCACACACCATAGA3’); p16^INK4a^ Forward (5’CTCAGCCCGCCTTTTTCTTC3’) and p16^INK4a^ Reverse (5’CGCCTTCGCTCAGTTTCTCATG3’); and β-actin Forward (5’ACCCCGTGCTGCTGACCGAG3’ and β-actin Reverse (5’TCCCGGCCAGCCAGGTCCA3’).

### 4.12. Statistical Analysis

Statistical analyses were carried out using SPSS 19.0 software (SPSS Inc., Chicago, IL, USA). Data are presented as the mean ± SD. Single factor ANOVA and the LSD test were used for comparison. Differences were considered significant at *p* < 0.05.

## 5. Conclusions

In summary, ginsenoside Rg1 improved the resistance of Sca-1^+^ HSC/HPCs in a mouse model of d-galactose-induced aging; this may be related to the inhibition of oxidative stress and excessive activation of the Wnt/β-catenin signaling pathway, reduction of DNA damage and restriction of the p16^Ink4a^-Rb and p53-p21^Cip1/Waf1^ pathways. This research provided an experimental basis for the mechanism of Sca-1^+^ HSC/HPC aging from the d-galactose-induced mouse model, and the application of ginsenoside Rg1 in postponing senility *in vivo*, which contributes to further study on the prevention and treatment of aging and degenerative diseases.

## Figures and Tables

**Table 1:** The levels of T-AOC, SOD, GSH-px and MDA in different mouse models (mean ± SD, *n* = 5, ^#^ *p* < 0.05 *vs.* the control group, * *p* < 0.05 *vs.* the d-gal model group, ^☆^ *p* < 0.05 *vs.* the d-gal + VitE group).

Group	T-AOC (U/mgprot)	SOD (U/ML)	GSH-px (U)	MDA (nmol/ML)
control group	8.38 ± 1.79	120.87 ± 5.56	460.94 ± 14.77	5.39 ± 0.46
d-gal group	3.83 ± 0.78 ^#^	83.19 ± 5.11 ^#^	247.97 ± 16.43 ^#^	11.69 ± 0.71 ^#^
d-gal + Rg1 group	6.03 ± 0.66 ^#,^*	97.25 ± 4.38 ^#,^*^,^^☆^	326.11 ± 20.07 ^#,^*	8.03 ± 0.21 ^#,^*
d-gal + VitE group	5.40 ± 1.12 ^#,^*	108.02 ± 4.71 ^#,^*	357.18 ± 14.67 ^#,^*	7.89 ± 0.24 ^#,^*

## Abbreviations

Sca-1	stem cell antigen-1
HSC/HPCs	hematopoietic stem/progenitor cells
d-gal	d-galactose
VitE	vitamin E
SA-β-Gal	Senescence β-Galactosidase
CFU-Mix	colony forming unit-mixture
ROS	reactive oxygen species
T-AOC	total anti-oxidant
SOD	superoxide dismutase
GSH-Px	glutathione peroxidase
MDA	malondialdehyde
GSK3β	glycogen synthase kinase 3β
APC	adenomatous polyposis coli
TCF	transcription T cell factor
LEF	lymphoid enhancer factor
AGEs	advanced glycation end products
4-HNE	4-Hydroxynonenal
8-OHDG	8-hydroxy-deoxyguanosine
MACS	magnetic-activated cell sorting
DCFH	dichlorodi hydrofluorescein diacetate
DDR DNA	damage response
